# Prefrontal connections express individual differences in intrinsic resistance to trading off honesty values against economic benefits

**DOI:** 10.1038/srep33263

**Published:** 2016-09-20

**Authors:** Azade Dogan, Yosuke Morishima, Felix Heise, Carmen Tanner, Rajna Gibson, Alexander F. Wagner, Philippe N. Tobler

**Affiliations:** 1 Laboratory for Social and Neural Systems Research, Department of Economics, University of Zurich, Blümlisalpstrasse 10, 8006 Zurich, Switzerland; 2 Division of Systems Neuroscience of Psychopathology, University Hospital of Psychiatry, University of Bern, Bolligenstrasse 111, 3000 Bern, Switzerland; 3 Japan Science and Technology Agency, PRESTO, 4-1-8 Honcho Kawaguchi, Saitama 332-0012, Japan; 4 Department of Experimental Psychology, University of Oxford, 9 South Parks RoadOxford OX1 3UD, England; 5 Swiss Finance Institute, University of Zurich, Walchestrasse 98006 Zurich, Switzerland; 6 Leadership Excellence Institute Zeppelin, Zeppelin University, Am Seemooser Horn 20, 88045 Friedrichshafen, Germany; 7 Swiss Finance Institute, University of Geneva, 42 Bd du Pont d’Arve1211 Geneva 4, Switzerland.

## Abstract

Individuals differ profoundly when they decide whether to tell the truth or to be dishonest, particularly in situations where moral motives clash with economic motives, i.e., when truthfulness comes at a monetary cost. These differences should be expressed in the decision network, particularly in prefrontal cortex. However, the interactions between the core players of the decision network during honesty-related decisions involving trade-offs with economic costs remain poorly understood. To investigate brain connectivity patterns associated with individual differences in responding to economic costs of truthfulness, we used functional magnetic resonance imaging and measured brain activations, while participants made decisions concerning honesty. We found that in participants who valued honesty highly, dorsolateral and dorsomedial parts of prefrontal cortex were more tightly coupled with the inferior frontal cortex when economic costs were high compared to when they were low. Finer-grained analysis revealed that information flow from the inferior frontal cortex to the dorsolateral prefrontal cortex and bidirectional information flow between the inferior frontal cortex and dorsomedial prefrontal cortex was associated with a reduced tendency to trade off honesty for economic benefits. Our findings provide a novel account of the neural circuitry that underlies honest decisions in the face of economic temptations.

Decisions concerning honesty are one of the most common moral decisions individuals make, not only in everyday situations but also in contexts that have great economic and political impact. For example, CEOs of large companies can, and did, in the case of Enron, WorldCom and many others, resort to dishonest practices, such as announcing higher earnings than the company actually achieved. Financial fraud (e.g., the cases of Bernard Madoff and Jerome Kerviel) and banking scandals (e.g., the LIBOR and FX market manipulations) have rocked the business world as well. Deceptive behavior can have devastating consequences for employees, shareholders, and the economy at large, as evidenced by the cases mentioned. Importantly, even before entering the realm of fraud, there is an area where moral judgments can play a big role. For example, the choice whether to report earnings that deviate from the true earnings with the intention of misleading stakeholders (i.e. manage earnings)^1^ is essentially a moral decision, given that the practice itself is legal, at least up to a point. Critically, CEOs are tempted to be dishonest because they can improve their personal finances considerably through bonuses tied to announced earnings or to share prices, which are, at least in the short run, positively affected by the announcement of higher earnings. Conversely, and surprisingly from the point of view of standard economics, some individuals are able to resist the financial temptation and remain honest. Moreover, whistleblowers who reveal misconduct risk careers and friendship, and journalists who report the truth about human right violations even risk their lives.

As exemplified with decisions to manage earnings, decisions about honesty often require weighing the benefits of an option against its costs. Thus, while most individuals value the mere act of telling the truth, they often also care about the consequences of their actions. Importantly, individuals differ in the extent to which they consider the economic consequences of honesty, which leads to individual differences in the tendency to tell the truth. When the economic costs of telling the truth are low, the benefits of being truthful outweigh the costs for many individuals. However, when these costs increase, some individuals no longer are willing to incur them, while others stand by their moral principles and continue to disregard the economic costs of doing so. Thus, individual differences in honesty should be more pronounced when the cost of telling the truth is higher. Interestingly, previous research has shown that honesty-related values that reflect individuals’ moral motives do indeed predict cost-dependent behavioral differences in honesty^2^. Accordingly, it is important to consider how individual differences in honesty motives affect the processing of economic costs because economic costs provide a strong and common incentive for dishonesty.

At the neural level, there is substantial evidence from recent neuroimaging studies that the prefrontal cortex is critically involved when individuals make decisions concerning honesty. Particularly the dorsolateral prefrontal cortex (DLPFC), the dorsomedial prefrontal cortex (DMPFC), the ventrolateral prefrontal cortex (VLPFC) and the inferior frontal gyrus (IFG) have been implicated in decisions concerning honesty^3^,^4^,^5^,^6^,^7^,^8^,^9^,^10^. However, given that these regions have different functions and are anatomically connected^11^, it is likely that decisions concerning honesty are implemented not by a single one of them, but through dynamic interactions among them. Therefore, understanding the neural mechanisms underlying decisions involving honesty makes it necessary not only to identify the activated brain regions in isolation, but also to illuminate how these regions flexibly interact with each other as a function of situation and honesty-related motives. This aim can be achieved by assessing changes in strength or direction of the functional coupling between brain regions^12^. A similar approach has revealed prefrontal interactions during other types of costly decisions (e.g., in value-based decisions involving effort)^13^. Here, we hypothesized that connectivity between prefrontal regions should be particularly pronounced in participants with strong honesty-related values when the economic costs of telling the truth are high. This hypothesis has not been investigated so far, even though previous behavioural research clearly showed that individual differences in honesty-related values exist and that economic costs of telling the truth are processed^2^,^14^. Indeed, previous research on the neural architecture of decisions concerning honesty has focused on activations^3^,^7^,^15^ rather than interactions between prefrontal regions. In the present study, we set out to close these gaps.

Our aim was to investigate how neural connectivity patterns change as a function of the economic costs associated with telling the truth and of individual differences in honesty-related values. We used functional magnetic resonance imaging (fMRI) to measure brain activations while participants made decisions concerning truthfulness. In each trial, participants had to decide between an honest and a dishonest option. Importantly, the dishonest option led to higher actual payoffs for the participant, such that choosing the honest option incurred an economic cost compared to choosing the dishonest option. Across trials we varied these costs, which in turn allowed for trading-off economic with honesty-related incentives. We investigated connection parameters (strength and direction of coupling between prefrontal regions) that reflect honesty-related values, and that change as a function of the costs of truthfulness.

## Methods

### Participants

Thirty-two subjects (14 female; aged 22.0 ± 2.8 (mean ± SD) years; range: 18–28) took part in the experiment. None of them had prior histories of neurological or psychiatric disorders and all had normal or corrected-to-normal vision. The study was approved by the Research Ethics Committee of the Canton of Zurich, and all subjects provided informed consent. The methods were carried out in accordance with the approved guidelines.

### Experimental design

#### Task instructions and payments

Participants performed a task that was adapted from a previously established behavioral paradigm^2^. They were first asked to read the instructions and answer control questions in order to ensure that they understood the tasks and the rules of the experiment. Before entering the scanner, the participants completed a series of practice trials. Inside the scanner, participants performed the truthtelling task and two control tasks that were matched to the truthtelling task in terms of potential earnings and costs but did not contain a honesty-related moral component (see Supplementary Methods: Experimental design and trial structure of the control tasks). Throughout the experiment, participants were placed in the situation of a CEO who had to make various incentivized decisions. Participants received a fixed basic payment of 25 Swiss Francs (CHF; at the time of the experiment, the exchange rate was about US $ 1 = CHF 0.91) and a variable additional payment, which corresponded to the summed earnings from five randomly selected trials per task and which was determined with the actual decisions made in these trials. The average variable payment was CHF 65.18.

**Figure 1:**
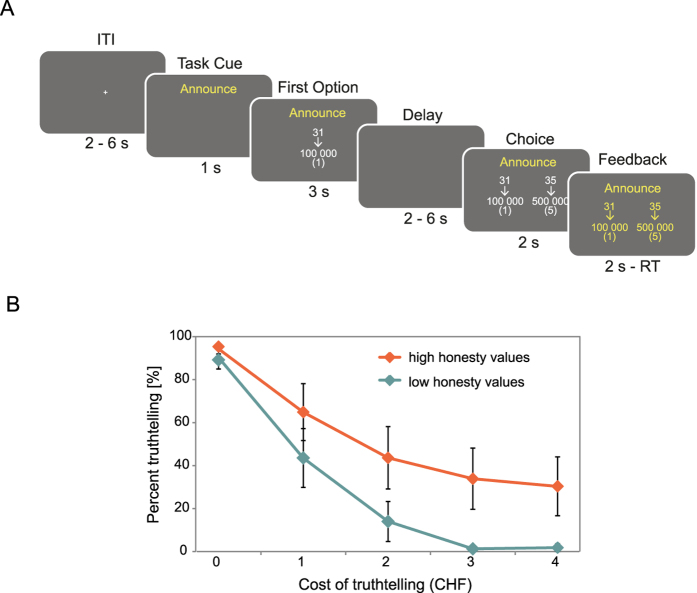
Experimental design and behavioral results. **(A)** Participants first viewed a fixation cross for a variable ITI of 2–6 s followed by the presentation of a cue (1 s) indicating that participants had to perform the truthtelling task. The first, variable option was then shown for 3 s together with the true earning (31 cents per share). Below the option, the CEO compensation (and the corresponding participant payoff in parentheses) was presented. The payoff of the truthful option varied between CHF 1 and 5. After an interstimulus interval (2–6 s) the second, constant option (5 CHF) was presented together with the first option. The second option was the false report (35 cents per share). Upon presentation of the second option, participants had 2 s to indicate their choice by performing a button press. As soon as they pressed a button, the color of the written text on the screen changed from white to yellow to indicate that a response had been recorded. **(B)** The difference in the percentage of truthtelling between individuals with stronger and weaker honesty-related values increased as a function of cost. When the economic costs of truthfulness were high, participants with stronger honesty-related values (upper tercile) were more honest compared to those with weaker honesty-related values (lower tercile), suggesting that honesty-related values are more important in determining truthful decisions when the costs of truthfulness are higher. Error bars indicate SEMs.

In each trial of the truthtelling task (Fig. 1A), participants had to announce the company’s earnings per share for the previous quarter. The participants were informed that the variable component of their payment as a CEO would be higher if they announced higher earnings. They were also told that the market and the shareholders anticipated earnings of 35 cents per share, but that the true earnings were 31 cents per share. Accordingly, participants could either announce 31 cents per share at an economic cost to them (see below) or announce earnings of 35 cents per share while knowingly remaining within legal accounting limits. Thus, participants had to choose whether to honestly announce true earnings or to lie and falsely report higher earnings. Importantly, compared to truthful reports, false reports led to higher actual payoffs for the participants, corresponding to a trade-off between honesty-related and economic incentives. This setup parallels a real-life conflict that CEOs face, as their variable compensation is often tied either to announced earnings directly or to stock price performance, which in turn depends on the earnings they announce. Thus, the CEO has economic incentives to behave dishonestly and boost earnings in order to increase his or her payout. Conversely, the CEO incurs real and personal economic costs when telling the truth. Importantly, a CEO’s inclination to tell the truth should decrease as the costs of telling the truth increase. Importantly, our experiment did not involve any aspect of altruism, reciprocity, guilt aversion, and strategic concerns, as there were no counterparties, and participants’ decisions had no impact on others. The primary candidate motivation for announcing the truth is, therefore, the intrinsic (moral or psychological) value of honesty.

The bonus to be gained from giving truthful reports varied across trials between CHF 100,000 and 500,000. The bonus to be gained from giving false reports was fixed to CHF 500,000. These amounts reflect the substantial monetary consequences that earnings management can have for CEOs of public corporations. Participants were informed that for their experimental payoff the CEO bonus would be converted into real money at a rate of CHF 100,000 = CHF 1.

#### Trial structure

In each trial of the task, participants first saw the variable, truthful and economically less beneficial option followed by the constant, but false and economically more beneficial option. The payoff of the first (honest) options varied between CHF 1 and 5 whereas the second (dishonest) option always led to a fixed payoff of 5 CHF. Thus, the cost of truthtelling varied between CHF 0 and 4. A previous study has shown that a range of CHF 0–1.20 is sufficient to induce variation in behavior in this truthtelling task^2^. Both task type and payoff-levels were randomly intermixed across trials during the experiment. Trials were separated from each other by an intertrial interval (ITI) with a mean duration of 4 s, varying from 2 to 6 s (Fig. 1A). Each trial started with the presentation of a cue (1 s) shown at the top of the screen that indicated which kind of task participants had to perform. Next, the first, i.e. variable, option was shown for 3 s in the center of the screen. Specifically, the true earning (31 cents per share) was presented together with the CEO compensation for announcing that option (participant payoff in parentheses). After an interstimulus interval consisting of a blank screen (mean of 4 s, varying between 2 and 6 s), the second, i.e. constant, option was presented, together with the first option. The second option was the false report (35 cents per share).

Participants were asked to make up their mind already during the presentation of the first option. This was possible as only the first option varied across trials, whereas the second option remained constant. The decision was conveyed during the presentation of the second option. To prevent motor preparation during presentation of the first option the two options were presented randomly either on the left or the right side of the screen during the response phase. Participants had 2 s to indicate their choice by performing a button press. When subjects pressed a button, the color of the written text on the screen changed from white to yellow to indicate that a response had been recorded. To keep them engaged throughout the task, in each trial in which participants failed to respond or responded too slowly, CHF 1 was deducted from their final monetary payment. These trials were repeated later. The average percentage of missed trials was 1.5% ± 0.6% (mean ± SEM; range 0–16%) with no significant difference between the tasks (F_(1,32)_ = 0.35, p = 0.70). Over the course of the experiment, each participant completed 75 trials of each task. Each of the five payoff levels was presented 15 times per task. Stimulus presentation and response recording were controlled using Cogent 2000 (Wellcome Department of Imaging Neuroscience, London, UK) as implemented in Matlab.

#### Measurement of honesty-related values

After scanning, we assessed participants’ honesty-related values^2^,^16^ that represent trait-like honesty motives based on moral beliefs rather than behaviour in the task. In the current study, we were specifically interested in honesty decisions under different cost situations. We therefore chose a questionnaire that has been shown to capture individual differences in honesty when there are economic costs of truthfulness^2^. This questionnaire was developed by Tanner and colleagues^16^,^17^. The full questionnaire consists of nine items, four of which aim to capture direct trade-off resistance to economic benefits, and five of which mostly capture indirect emotional responses against violations of honesty. The two scales were validated and shown to have good internal consistency (each Cronbach’s α higher than 0.79^17^; Cronbach’s α of the direct scale of 0.81^16^; combined Cronbach’s α of 0.86^2^). Although earlier work had shown that both subscales individually and the combined scale explain resistance against economic incentives to lie, our preliminary analysis indicated that the direct scale (four items) would be, as expected, particularly effective in capturing direct economic trade-off resistance. These four items read as follows:

*CEOs have an opportunity to modify information in the reports they provide to their shareholders. Some view such modification as a violation of truthfulness, others regard it as acceptable protection of personal interests. What do you think about the value of truthfulness in such a situation?*

#### Truthfulness is something

1. *That one should not sacrifice, no matter what the (material or other) benefits*.
2. *For which I think it is right to make a cost-benefit analysis*.
3. *That cannot be measured in monetary terms*.
4. *About which I can be flexible if the situation demands it*.

The questionnaire examined how much importance participants attributed to specific features of honesty versus economic trade-offs (such as trade-off reluctance, unwillingness to sacrifice honesty as a value, or incommensurability). In line with our research question on how agents differ in honest decision-making at high cost levels, the scale captures the extent to which individuals consider honesty as protected against trade-offs or indeed “sacred”^16^,^18^.

For every item, participants used a seven-point scale ranging from 1 (strongly disagree) to 7 (strongly agree). For inverted items we recoded answers such that higher numbers reflected higher values. Based on the mean across the items of the questionnaire, we then constructed an index of the degree of honesty-related values. For ease of interpretation of the empirical results, we changed the scale to range from 0 (for an individual with minimum honesty-related values) to 6 (for an individual with maximum honesty-related values).

Preliminary analyses of another study (data not shown here) indicate that, while other individual-specific measures (such as the HEXACO honesty-humility scale^19^, moral identity^20^, and social value orientation^21^) correlate with our measure of honesty-related values, only honesty-related values predict honest behavior in a cost-dependent manner. Earlier work using the same experimental paradigm indicates that even controlling for social desirability, the combined honesty scale explains truthfulness and reactions to economic costs^2^ (while social desirability explained neither truthfulness nor reactions to economic costs).

### Behavioral analysis

The effect of honesty-related values on choice behavior was analyzed using a logistic regression analysis. The dependent variable corresponded to the decision in a given trial. This was coded as a binary variable that took on the value of 1 if participants chose the honest option, and the value of 0 if participants chose the dishonest option. The independent variables (regressors) in the model included cost-level, honesty-related values, and their interaction, while controlling for gender and age. To determine the probability *P* of choosing the honest option, we used a logit model as follows:
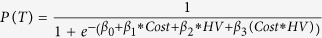
where
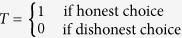
and *Cost* is the economic cost of telling the truth, *HV* denotes honesty-related values, and (*HV* * *Cost)* the interaction term between economic cost and honesty-related values.

### fMRI Data Acquisition

fMRI was performed on a Philips Achieva 3T whole-body scanner equipped with an eight-channel head coil (Philips Medical Systems, Best, The Netherlands) at the Laboratory for Social and Neural Systems Research, University of Zurich. We acquired gradient-echo T2*-weighted echoplanar images (EPIs) with blood-oxygen-level-dependent (BOLD) contrast (slices/volume, 37; repetition time, 2 s). Participants each completed five sessions of the experiment in the scanner, with short breaks between each session. 315–360 volumes were collected per session (the variation was due to individual differences in the number of repeated trials) along with 5 “dummy” volumes at the start of each session to allow for magnetization to stabilize to a steady state. Scan onset times varied randomly relative to stimulus onset times. Volumes were acquired at a 15° tilt to the anterior commissure-posterior commissure line, rostral > caudal. Imaging parameters were the following: echo time, 30 ms; field-of-view, 220 mm; in-plane resolution, 2.75 mm; slice thickness, 3 mm; interslice gap, 0.5 mm. A T1-weighted structural image was also acquired for each participant. These high-resolution T1-weighted structural scans were coregistered to their mean EPIs and averaged to permit anatomical localization of the functional activations at the group level.

### fMRI Data Analysis

fMRI data processing and statistical analyses were carried out using statistical parametric mapping (SPM8; Wellcome Department of Imaging Neuroscience, London, UK). Data preprocessing consisted of realignment, slice-time correction, coregistration, segmentation, spatial normalization using the DARTEL toolbox and smoothing using a Gaussian kernel with a full width at half maximum of 10 mm.

#### Identification of seed regions

To identify our seed regions for the psycho-physiological interaction (PPI) analyses^12^, we focused on regions where cost-related activity increases with individual propensity of truthtelling. Specifically, we estimated a GLM that included regressors for the presentation of the first option of each task. These three regressors were defined irrespective of actual decisions and modeled as stick functions with duration of 0 s (for neural activity reflecting choice behavior see Supplementary Results: Choice-dependent neural activity). For each of the regressors we included a parametric modulator (PM) capturing the variable cost-level (PM cost). We also included regressors of no interest consisting of the onsets of decision time and the missed trials for each task. These regressors were convolved with the canonical hemodynamic response function. Participant-specific movement parameters were also included. The main effect for the cost of truthtelling was computed on the single-subject level by performing a one-sample t-test for the parametric modulator of the truthtelling task. The resulting contrast image was taken up to the group-level, where we used correlations with participant-specific percentage of truthtelling to identify the seed regions for the PPI analyses.

#### Psycho-physiological interaction (PPI) analyses

To test for coupling differences due to variations in cost-level and honesty-related values we performed PPI analyses^12^ using the generalized PPI method^22^.

Note that the regressors in the PPI were defined irrespective of actual decisions. We focused on high-cost versus low-cost trials because in high cost situations the potential tradeoff between economic costs and honesty-related values is more pronounced than in low cost situations. For each subject, the average time series was extracted from the DLPFC and DMPFC seed regions where activity for cost of truthtelling correlated significantly with individual percentage of truthtelling (see Results; Seed identification: Cost-related activations in the DLPFC and the DMPFC). These time series served as physiological regressors in two separate first-level general linear models for the PPI analyses. Moreover, each model included two psychological regressors: high cost, i.e. cost levels 3 and 4 and low cost, i.e. cost levels 1 and 2 (this allowed us to compare high costs, where choices were affected by honesty-related values, with low costs, where choices were similar irrespective of honesty-related values of individual participants). In addition, two PPI regressors were created by multiplying the time series with the two psychological regressors, respectively. Thus, the parameter estimates of the two PPI regressors reflect the correlation between activity in the DLPFC/DMPFC and activity in each voxel during high or low cost trials. We then computed for each participant the contrast between the two PPI regressors (high vs. low cost) and entered the resulting contrast images into a second-level correlation analysis with individual honesty-related values.

Correction for multiple comparisons (family wise error (FWE); p < 0.05) was performed in the whole brain at the cluster-level (cluster-inducing voxel-level threshold: p < 0.005), except for supplementary control analyses. All reported coordinates (x, y, z) are in MNI space.

#### Dynamic causal modelling (DCM)

To characterize prefrontal information flow in individuals with strong honesty-related values, we examined the coupling direction between the IFG and the DLPFC and the IFG and the DMPFC by using DCM^23^ in the sixteen participants with honesty-related values above the median (median = 3.88). We were specifically interested in these participants as the PPI analysis revealed stronger cost-related connectivity with the IFG only in these subjects. We aimed to understand how specifically high costs affect the coupling direction between these regions. For each participant, we extracted activation time courses (principal eigenvariates) from a 4 mm-radius sphere around the peak activations identified in the preceding analyses. These regions were the DLPFC (−30, 56, 26), DMPFC (−6, 16, 44) and the IFG (−44, 28, 28). The principal eigenvariates were adjusted for the F-contrast capturing unspecific effects of interest.

At the outset, we ensured that all potential DCMs were anatomically plausible. This was indeed the case, as direct connections between the three regions (DLPFC, DMPFC, and IFG) have been demonstrated in humans and non-human primates^24^,^25^,^26^,^27^,^28^,^29^,^30^. Further, we included only models that contained a plausible information flow based on the combinatorial variations in driving inputs and modulatory connections. Hence, we ensured that every region receives either a driving input or input from another region and thus is not isolated from the flow of information. For example, we did not include a model (Supplementary Fig. S5) in which the driving input enters only the DLPFC and the modulation of connectivity is from the IFG to the DLPFC and the DMPFC but not from the DLPFC to the IFG, because modulation from the DLPFC to the IFG should be present if the information entering only the DLPFC is to exert any effect. Thus, the models in our DCM analysis were based on criteria of functional and anatomical plausibility as well as internal consistency. As a result, we were left with 41 models. We categorized these 41 DCMs into nine different families, with each family containing between two and seven models (depending on how many models ensured plausible information flow). The categorization into families was based on connectivity patterns between the DLPFC and the IFG on the one hand and the DMPFC and the IFG on the other hand. The modulatory effect (modelled as a stick function) consisted of high cost conditions and the families differed in the way by which this modulatory effect influenced the connections between the DLPFC and the IFG (either from DLPFC to IFG, the IFG to the DLPFC or bidirectional) and the DMPFC and the IFG (either from DMPFC to IFG, the IFG to the DMPFC or bidirectional). Note that the modulatory effect consisted of high cost conditions and that the DCMs were not based on actual decisions. Within each family the models varied in terms of the driving input. Driving inputs represent extrinsic parameters that change the neuronal state of brain regions within a model (here, visual presentation of the first option). We specified driving inputs either into the DLPFC, the DMPFC, the IFG or any combination of these regions. We identified the most likely family of models using Bayesian model selection^31^,^32^. This allowed us to compare the model families based on their exceedance probabilities, a measure of whether a model family is more likely to be correct than all other model families. Lastly, we used Bayesian model averaging^33^ for the most likely family of models to compute the magnitudes of coupling parameters.

## Results

### Behavioral results

#### Average behavior

In the truthtelling task, participants on average told the truth in less than half of the trials (39.8% ± 5.1%, mean ± SEM), suggesting that the dishonest option was the default choice in this task. Participants also showed a wide variation in their choices with some participants choosing to tell the truth in all trials and others doing so in very few trials (range 6.7–100%). Importantly, we also observed variation in their choices with respect to the economic cost of truthtelling: 86.9% ± 3.9% in the zero cost condition, 50.4% ± 8.0% in the CHF 1, 30.8% ± 7.3% in the CHF 2, 16.7% ± 6.1% in the CHF 3, and 14.4% ± 5.1% in the CHF 4 condition. The mean switching point (point at which participants were indifferent between the honest and dishonest option) was CHF 1.5 ± 0.25 (mean ± SEM).

#### Honesty-related values predict decisions in different cost situations

To capture individual honesty motives, participants completed a questionnaire that assessed their honesty-related values, which measures the extent to which honesty values are regarded as protected, that is, as priceless and not subject to trade-offs (see Methods: Measurement of honesty-related values). We expected that strong honesty-related values should allow participants to more often resist the temptation of not being truthful for economic benefits, particularly when the economic costs for telling the truth were high. Thus, compared to weaker honesty-related values, strong honesty-related values should lead to relatively more honest decisions in higher cost conditions. We assessed this hypothesis in an interaction analysis using our honesty-related value questionnaire. In agreement with the hypothesis, we observed a positive interaction between honesty-related values and economic costs of truthfulness (β = 0.28 ± 0.13, t = 2.15, p < 0.05). In other words, when economic costs of truthfulness were high, participants with stronger honesty-related values chose the truthful option more often compared to participants with weaker honesty-related values (Fig. 1B; see Supplementary Fig. S2 and Supplementary Results: Relation of honesty-related values to behavior in control tasks and to response times). Taken together, these data suggest that strong honesty reduced (but did not eliminate) participants’ willingness to trade off honesty against economic costs.

### Neuroimaging results

#### Seed identification

**Figure 2:**
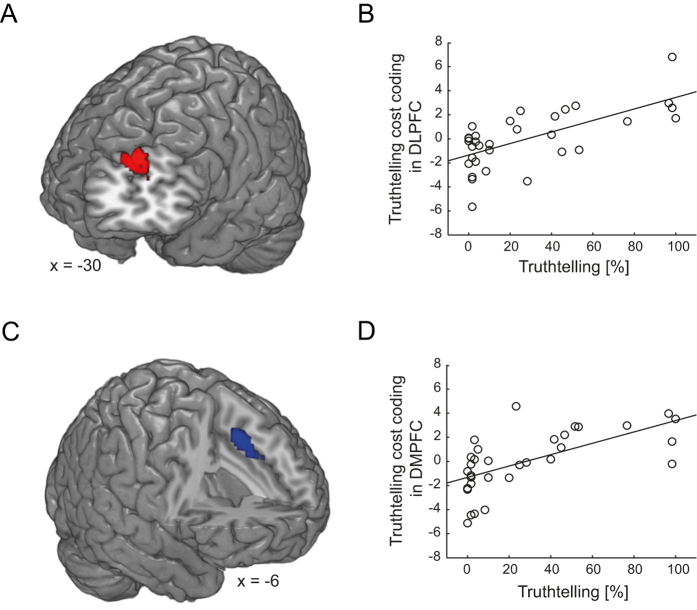
Activity in DLPFC and DMPFC (seed regions for Fig. 3) reflects truthtelling costs with increasing propensity to be honest. (**A**,**B)** DLPFC responses to the cost of truthtelling increased with the individual percentage of truthtelling (−30, 56, 26; t_(30)_ = 4.92; p < 0.05, whole-brain FWE cluster-level corrected). **(C**,**D)** DMPFC responses to the cost of truthtelling increased with the individual percentage of truthtelling (−6, 16, 44; t_(30)_ = 4.48; p < 0.05, whole-brain FWE cluster-level corrected). Throughout, activations are displayed at the uncorrected level of p < 0.001 for visualisation purposes.

Cost-related activations in the DLPFC and the DMPFC. Based on the behavioral findings, we asked how stronger honesty-related values affect neural interactions, specifically when honest behavior is more costly. To do so, we first identified activation-based seed regions where coding of the economic costs of telling the truth related to propensity for telling the truth. Thus, we searched for brain regions where activation related to cost-level (parametric modulator) changed as a function of percent truthtelling (note that the regressors were defined irrespective of actual decisions; for neural activity reflecting choice behavior see Supplementary Results: Choice-dependent neural activity). As a trade-off between moral and economic incentives can arise only when at least some economic costs are incurred for telling the truth, we calculated the percentage of truthtelling without taking the zero cost condition into account. We found that neural coding of the cost of truthtelling increased with the individual percentage of truthtelling in the DLPFC and DMPFC (Fig. 2A,B; DLPFC: −30, 56, 26; t_(30)_ = 4.92; Fig. 2C,D; DMPFC: −6, 16, 44; t_(30)_ = 4.48; both p < 0.05, whole-brain FWE cluster-level corrected; see Supplementary Methods (Control analyses for the identification of the seed regions for the truthtelling PPI analyses) and Supplementary Results (Control analyses: difficulty) for control analyses). In other words, with increasing cost of telling the truth, participants show stronger activation in the DLPFC and DMPFC the more often they make honest decisions. We used these two clusters as seed regions for the subsequent PPI analyses (for the seed identification relevant for comparing the connectivity pattern of the truthtelling task with that of the control tasks see SI Methods: Identification of seed regions for specificity PPI analysis and SI Results: Specificity PPI analyses).

#### DLPFC-IFG and DMPFC-IFG interactions depend on cost levels and honesty-related values

**Figure 3:**
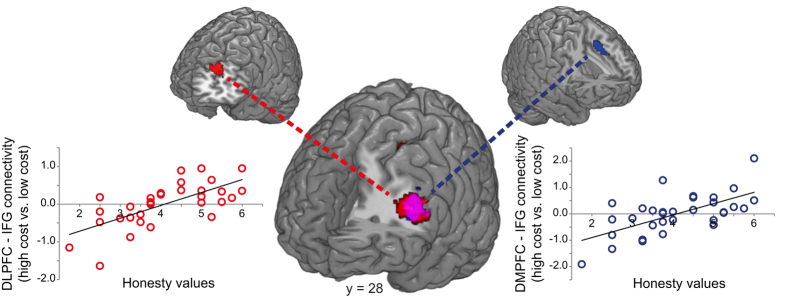
DLPFC-IFG and DMPFC-IFG interactions increase with truthtelling costs particularly in individuals with stronger honesty-related values. Functional connectivity between the honesty-related seed regions DLPFC and DMPFC (shown at smaller scale) and the IFG (DLPFC-IFG: −46, 28, 30; t_(30)_ = 4.56; DMPFC-IFG: −44, 28, 28; t_(30)_ = 5.12; both p < 0.05, whole-brain FWE cluster-level corrected) was increased in high compared to low cost conditions as honesty-related values increased. Voxels in red show correlation with the DLPFC seed, voxels in blue show correlation with the DMPFC seed, and voxels in purple show the overlap between the correlations of the two seed regions. The left graph shows individual connectivity strengths for the DLPFC-IFG interaction and the right graph shows individual connectivity strengths for the DMPFC-IFG interaction. Please note that this figure illustrates the results of a PPI analysis over all subjects. The y-axis shows the coupling difference for high-low cost conditions and the negative values in low honesty value individuals are a consequence of this fact. In other words, for these individuals coupling is higher for low cost than high cost conditions. However, in all conditions, coupling tended to be positive (Supplementary Fig. S4).

In the next step, we tested whether participants with stronger honesty-related values exhibit a specific prefrontal connectivity pattern with regard to the economic cost of honesty. We therefore investigated whether the regions identified to process costs in an individual-specific manner (DLPFC and DMPFC; see above) interact differently with other regions of the decision network as a function of cost and honesty-related motives. Accordingly, we performed two PPI analyses with either the DLPFC or the DMPFC as seed region (see Methods). We then performed a second-level correlation analysis with the individual PPI contrast images (PPI regressor of high vs. low cost) with our participant-specific measure of honesty-related values. We found that functional connectivity between the DLPFC and the IFG as well as between the DMPFC and the IFG differed significantly as a function of cost-level and honesty-related values (Fig. 3; DLPFC: −46, 28, 30; t_(30)_ = 4.56; DMPFC: −44, 28, 28; t_(30)_ = 5.12; p < 0.05, both whole-brain FWE cluster-level corrected; additional whole-brain cluster-level corrected activation was found in the parietal cortex: −46, −32, 38; t_(30)_ = 4.29). Specifically, both DLPFC and DMPFC showed stronger functional connectivity with the IFG in high cost conditions compared to low cost conditions as honesty-related values increased. We found the same results when we included percent truthtelling as a covariate of no interest (see Supplementary Results (Control analyses: PPI with control variables as covariates of no interest); also see Supplementary Results: Specificity PPI analyses, where we compared the connectivity pattern of the truthtelling task with that of the control tasks). Thus, stronger honesty-related values tightened the coupling between prefrontal decision regions particularly when the costs for telling the truth were high.

#### Directionality of prefrontal connections

**Figure 4:**
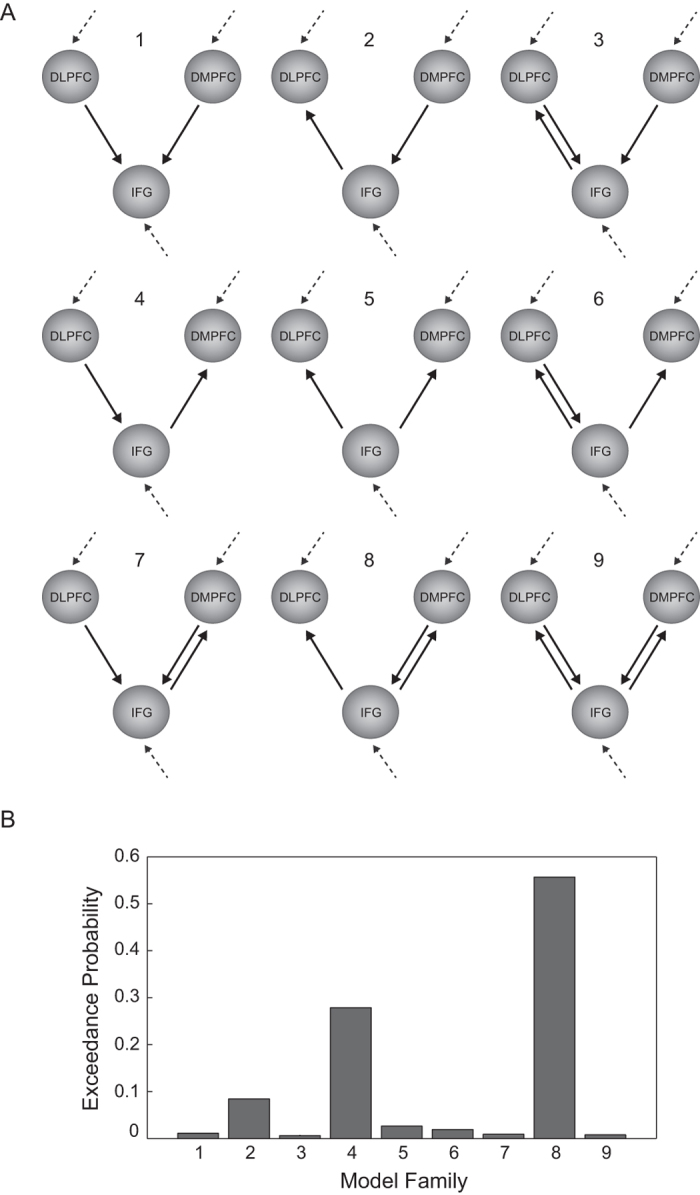
DCM models and results of Bayesian model selection. **(A)** Illustration of nine alternative model families with changes in connectivity modulated by high cost. Arrows represent potential modulation of coupling between the DLPFC and IFG and the DMPFC and the IFG. Models within a family differed in terms of driving input region (grey dashed arrows: driving inputs could be either into the DLPFC, the DMPFC, the IFG or any combination of these regions). **(B)** Bar graph showing the exceedance probability for each model family. The labels on the x-axis correspond to the numbers assigned to each model family in A.

Our PPI results show that in individuals with stronger honesty-related values the DLPFC and DMPFC are more strongly coupled with the IFG when honesty is more costly. In order to better understand the nature of these couplings, in the final step of our analysis, we examined the directionality of the connections by performing a DCM analysis. As the prefrontal connections were more pronounced for participants with strong honesty-related values and particularly during high cost conditions, we focused our DCM analyses on these participants and investigated the modulatory effect of the high cost condition (for a DCM analysis on all participants, Supplementary Fig. S6). In particular, we specified and estimated nine model families that were grouped based on how the high cost condition modulated the connection between the DLPFC and IFG on the one hand and the DMPFC and IFG on the other hand (Fig. 4A). We used Bayesian model selection to identify the most probable family of models. The most likely model family (family 8 in Fig. 4A; exceedance probability = 0.56; twice as high as the second preferred model with an exceedance probability of 0.28) was the one with a unidirectional connection from the IFG to the DLPFC and a reciprocal connection between the DMPFC and IFG (Fig. 4B). Finally, we used Bayesian model averaging to estimate the coupling parameters of the most likely model family. We found that the coupling from the IFG to the DLPFC was positive (0.11 Hertz; Hz). This indicates that an increased rate of IFG activity under the high cost condition results in an increase of DLPFC activity, suggesting that in high cost situations the activity in the DLPFC is more responsive to signals in the IFG. The bidirectional DMPFC-IFG coupling showed a positive modulation for the connection from the DMPFC to the IFG (0.11 Hz) and a negative one for the connection from the IFG to the DMPFC (−0.23 Hz).

## Discussion

In the present study, we used two complementary methods, PPI and DCM, to investigate how task-related connectivity relates to honesty costs and honesty-related values during truthtelling decisions. Our PPI results show that in high, compared to low-cost situations, the DLPFC and DMPFC are more strongly coupled with the IFG in individuals with stronger honesty-related values. Thus, individuals with stronger honesty-related values exhibit prefrontal interactions when the costs of honesty are high. Furthermore, our DCM results specified the direction of these interactions by showing that the coupling in participants with strong honesty-related values was best explained by a model in which the DLPFC receives input from the IFG and the DMPFC and IFG are bidirectionally coupled. Together, the findings provide novel insights into how prefrontal connectivity underpins individual variability in honesty-related values.

In line with previous behavioral research^2^, we find that both personal factors (honesty-related values) and situational factors (cost-level) determine whether individuals behave honestly. More specifically, when the economic costs of truthfulness were high, participants with stronger honesty-related values chose the truthful option more often compared to participants with weaker honesty-related values. This suggests that honesty-related values are instrumental in driving truthful decisions particularly when truthfulness is more costly.

Our measure of honesty-related values assesses the extent to which individuals regard honesty as protected and not subject to trade-offs. Thus, the present findings should be interpreted only with respect to this type of honesty-related values. Indeed, different dimensions of honesty-related values exist^2^. Interestingly, supplementary analyses of our data suggest that the cost-dependent prefrontal connectivity modulations are specifically related to honesty values that are expressed by trade-off reluctance as compared to honesty values captured by emotional responses to dishonesty (see Supplementary Results: Comparison between different types of honesty values), but further types of honesty-related values remain to be investigated.

The DLPFC, DMPFC and IFG have been consistently implicated in cognitive control and response inhibition^11^,^34^,^35^,^36^ and previous studies have suggested that deceptive behavior engages the control network^3^,^7^,^9^,^15^. However, other studies show that the engagement of control regions is also crucial in making honest decisions^4^,^37^. In this regard, it is important to remember that our study investigated primarily the coding of economic costs of honesty during honesty-related decisions irrespective of whether these decisions resulted in truthtelling or lying. Still, one possible interpretation of our findings could be that in high compared to low cost conditions increased prefrontal connectivity represents an active control mechanism that helps individuals with strong honesty-related values to resist temptations for dishonest behavior. In line with this interpretation, recent evidence shows that active cognitive control can indeed support honest behavior^38^,^39^,^40^, that areas of the control network are more strongly activated during truthtelling as compared to lying^37^, and that lesions in prefrontal control regions reduce honesty^41^. This view is further supported by the enhanced prefrontal connectivity during low rather than high cost conditions in individuals with weak honesty values (Supplementary Fig. S4). These individuals are more likely to perceive a trade-off between telling the truth and its economic costs in low cost than in high cost conditions. By contrast, in high cost situations they are less likely to perceive such a trade-off. Together, individuals appear to flexibly deploy prefrontal control mechanisms to potentially increase honesty as a function of both personality (honesty values) and situational factors (cost level), when called for by perceived trade-offs. Importantly, the present study for the first time reveals how core regions of the control network interact with each other as a function of economic costs of honesty.

It has been proposed that strong moral values can be derived from rules that prohibit certain actions^42^. Such rules come in different forms^18^. In our case, for example, the rule comes in the form of not to trade-off honesty for economic benefit. Indeed, the measure we use for honesty-related values focuses on this idea of trade-off resistance. On a very basic level, these rules might be stored as semantic knowledge, which is retrieved when individuals are faced with decisions that are related to those rules. Interestingly, the IFG has been associated with semantic rule retrieval and processing^43^,^44^,^45^. Our PPI analyses showed that high truthtelling costs increased the connection between the IFG and the DLPFC and the DMPFC. Thus, the IFG could provide input that represents semantic rules, which specifically in high cost situations need to be retrieved in order to incline participants towards honesty irrespective of consequences. In agreement with the proposed role of the IFG, activation in the IFG has been found to be stronger when individuals refuse to accept money for a change in their previously reported moral views compared to when they accept^46^ (see also^47^). Our results reveal that the DLPFC-IFG and the DMPFC-IFG connectivity in individuals with strong honesty-related values is enhanced specifically when high economic costs are involved and thus strong control mechanisms are needed to protect the honesty-related values.

High costs modulated the bidirectional DMPFC-IFG coupling by facilitating the excitatory connectivity between the DMPFC and the IFG and increasing the inhibitory connectivity between the IFG and the DMPFC. This means that in participants with high honesty-related values, honesty in high cost situations may be achieved by a combination of unidirectional connections between IFG and DLPFC and bidirectional connections between the DMPFC and the IFG. Given the proposed distinctive roles of these regions in cognitive control^36^,^48^,^49^,^50^, one could speculate that during high cost conditions at first the DMPFC specifies and monitors the required control by communicating with the IFG that retrieves the moral rule, and the DLPFC then receives input from the IFG to actively execute control.

Our supplementary findings not only confirm that individual differences in honesty-related values predict the strength of DLPFC-IFG and DMPFC-IFG connectivity in the honesty task, but also suggest that these interactions are not specific for decisions concerning honesty (see Supplementary Results: Specificity PPI analyses). We thereby re-visit the question of whether moral decisions are represented by domain-specific or domain-general neural mechanisms^51^,^52^ and for decisions concerning honesty our results support domain-generality^53^.

Although our truthtelling task mirrors one important real-life conflict CEOs face on a regular basis, it is equally important to note that in real life there are also other factors that may play a role. The advantage of the experiment is that we can exclude them. For example, our participants did not need to consider possible risks of telling a lie. Even though CEOs in the real world as well as in our task can manage earnings legally within certain accounting limits, they still face the risk of losing their job if their dishonesty is revealed. This risk would reduce the propensity of CEOs lying in the real world compared to our experiment. By contrast, CEOs but not our participants could have the belief or at least hope that the truth will not be revealed to others, which would increase the propensity of CEOs lying in the real world compared to our experiment. By extension, as participants’ decisions were revealed to the experimenters in our study, they may have perceived choosing the dishonest option as involving a loss in reputation, which in turn could have mediated their decisions. Although we cannot completely exclude it, this possibility is unlikely given that participants were told that choosing the dishonest option would be within legal accounting limits. Still, it is worth keeping in mind that in practice, there are also other motives for managing earnings, for example, competitive pressures among firms.

Another limitation of this study is that we collected only a limited number of other individual-specific measures. This study cannot shed light on how our measure of honesty-related values is related to other personality traits. Our supplementary findings (Supplementary Results: Control analyses with demographic variables and other individual-specific measures) suggest that the variables that were assessed neither correlate with our measure of honesty-related values, nor do they change the cost-dependent relation to behavior when included as control variables. Moreover, preliminary analyses of another study (see Methods: Measurement of honesty-related values) indicate that only honesty-related values as compared to other individual-specific measures predict honest behavior in a cost-dependent manner. This further corroborates that our measure of honesty-related values is particularly well-suited to capture cost-specific changes in decisions concerning honesty. Finally, we acknowledge that collecting questionnaire data after the experiment could have biased participants to respond in a way that matches their behavior in the truthtelling task. We chose this order to prevent the opposite bias, i.e. that participants behave in a way that matches their previous responses in the questionnaire. Although we cannot completely rule out the possibility that questionnaire responses were influenced by experimental decisions, several considerations suggest that it is unlikely. First, responses on our questionnaire are not significantly different in participants who never performed the experiment compared to participants who answered the questionnaire after the experiment^2^. Second, different participants give similar responses to the questionnaire irrespective of whether they complete the questionnaire two weeks before the experiment (unpublished pilot data) or as in the current study directly after the experiment (t_(84)_ = 0.78; p = 0.44). Third, honesty-related values did not correlate with tendencies for self-deception or impression management (see Supplementary Results: Control analyses with demographic variables and other individual-specific measures).

In conclusion, our results provide the first evidence that different prefrontal regions interact during decisions concerning honesty. These interactions occur in a flexible manner, leading to connectivity patterns that are situation-specific and depend on honesty-related motives. In particular, individual differences in honesty-related values modulate DLPFC-IFG and DMPFC-IFG connectivity in high cost situations. Our findings highlight the relevance of studying prefrontal connectivity and individual differences in how people value the appropriateness of accepting economic benefits in exchange for dishonesty. By extension, prefrontal coupling patterns could be responsible for predisposing some CEOs to immoral behavior for high personal gains. Our findings might also offer a framework to study the more extreme forms of immoral and deceptive decision making in psychopathy and antisocial personality disorder.

## Additional Information

**How to cite this article**: Dogan, A. *et al*. Prefrontal connections express individual differences in intrinsic resistance to trading off honesty values against economic benefits. *Sci. Rep*. **6**, 33263; doi: 10.1038/srep33263 (2016).

## Supplementary Material

Supplementary Information
