# Supplementary material for: Prefrontal connections express individual differences in intrinsic resistance to trading off honesty values against economic benefits
Source: Sci Rep. 2016 Sep 20;6:33263. doi: 10.1038/srep33263 (PMC5028845; doi:10.1038/srep33263)
Supplement: Supplementary Information [file srep33263-s1.pdf]

Supplementary Information

Prefrontal connections express individual differences in intrinsic resistance to trading off honesty values against economic benefits

Azade Dogan, Yosuke Morishima, Felix Heise, Carmen Tanner, Rajna Gibson, Alexander F. Wagner, Philippe N. Tobler

Supplementary Methods

Experimental design and trial structure of the control tasks

As in the truthtelling task, in the two control tasks (the effort and the valuation task) the decisions the participants made as a CEO affected stock value and therefore their compensation. In the effort task, participants chose how much work to invest as a CEO. The participants were told that the more they worked the more the company would profit and the more they would earn as a CEO. Specifically, in each trial participants decided how many simple math problems to solve after the main experiment. The participants had to solve these math problems outside of the scanner, using a computer screen. They chose between solving only one problem or five problems, which would require more effort and take five times longer than solving one problem. Yet, the five problems option typically led to a higher actual payment for the CEO (CHF 500,000) than the one problem option (CHF 100,000 - 500,000), resulting in a trade-off between economic incentives versus effort and/or time. For brevity, we refer to this task as the "effort"-task. In the valuation task, participants chose between two projects to invest in. The two projects were similar in their properties (e.g. risk-free and realized after the same delay) but differed in their profit and accordingly the CEO compensation. The high value project (labelled project "XIR" in the task) typically led to a higher actual payment for the CEO (CHF 500,000) than the low value project (labelled "ZEM"; CHF 100,000 - 500,000). There was no trade-off in this control task as there were no costs for choosing the high value option. However, the variable economic value difference between the two options was equivalent to that of the other two tasks, that is, in all tasks and trials participants decided between a variable (CHF 1-5) and a fixed payoff option (CHF 5).

The trial structure of the control tasks was similar to that of the truthtelling task (Supplementary Fig. S1). First, a cue indicated which task participants had to perform. The cue

"Calculate" referred to the effort task and "Perform" to the valuation task. Next, the first, i.e. variable, option was shown for 3 s in the center of the screen. In the effort task, the first option was one calculation and, in the valuation task, the low value project. Below the option, the CEO compensation for that option was indicated together with the corresponding participant payoff in parentheses. After an interstimulus interval consisting of a blank screen (mean of 4 s, varying between 2 and 6 s), the second, i.e. constant, option was presented, together with the first option. The second option was five calculations in the effort task, and the high value project in the valuation task.

Measurement of effort-related values

Similarly to the questionnaire measuring honesty-related values, participants filled in a questionnaire assessing effort-related values, where subjects had to indicate their views about spending more time to solve more math problems and earning more money.

The math problems are examples of decision situations in which individuals have to weigh between time and money. Some people easily give up time in order to earn more money, however others don't. What do you think about the value of time in such a situation?

Time is something

1) *… that one should not sacrifice, no matter what the (material or other) benefits.*
2) *… for which I think it is right to make a cost-benefit analysis.*
3) *… that cannot be measured in monetary terms.*
4) *about which I can be flexible if the situation demands it.*

Supplementary fMRI Data Analysis

Analysis for the identification of choice-dependent neural activity. To identify neural activity related to actual behavior we estimated a separate GLM. For this analysis we included twenty-one participants who showed both honest and dishonest behavior. Specifically, this GLM modeled each decision in separate regressors depending on individual choice (honest choice and dishonest choice). We also included regressors for the control tasks (low value project choice, high value project choice, low

effort choice, and high effort choice). The regressors were modeled at the presentation time of the (first) option and with a variable duration until the time of the decision. We included regressors of no interest consisting of the onsets of the motor response, the missed trials and participant-specific movement parameters. A linear contrast of regression coefficients of honest vs. dishonest (and vice versa) was computed at the single-participant level and then taken to group-level analyses where we used one-sample t-tests to identify differential activations.

Control analyses for the identification of the seed regions for the truthtelling PPI analyses. To control for decision difficulty, we estimated a GLM that was a variant of the one described in the main text and included a measure of individual, trial-specific difficulty as an additional parametric modulator (PM difficulty). For each task we first determined the point at which each participant was indifferent between the economically more costly option and the less costly option. For example, if in the truthtelling task the participant switched from honest to dishonest decisions between a cost of 1 CHF and 2 CHF, the indifference point would be 1.5 CHF, indicating a 50% probability of choosing either option. At this point, decisions are most difficult. Based on this indifference point, the difficulty of each trial was calculated as the distance between the trial's cost-level and the indifference point. These difficulty measures were determined for each task separately and entered as a second parametric modulator after the parametric modulator of cost, without using orthogonalization. Thus, the two parametric modulators competed independently for how well they explained brain activity. For this analysis we included twenty-six participants for the truthtelling task and twenty-five participants for the effort task. These participants chose both the costly as well less costly option over the course of the experiment and we were therefore able to determine the indifference point. Single-subject contrasts were calculated for PM cost vs. PM difficulty.

Identification of seed regions for specificity PPI analysis. A central question in the study of morality concerns the extent to which moral and non-moral decision making relies on similar or different neural mechanisms. While it has been argued that moral decision making relies on common ("domain-general") rather than dedicated ("domain-specific") neural mechanisms[1–3], there have also been

proposals that morality forms a domain of its own[4]. To address this issue, we asked whether we find connectivity patterns that are specific for honesty as compared to control decisions. In order to ask this question in an unbiased manner, the seed regions need to be related to both types of decisions. We therefore aimed to identify seed regions that show cost-related activity increases common to the truthelling and control tasks. Using the GLM described in the main text, main effects for the cost of effort and valuation were computed on the single-subject level by performing separate t-tests for the parametric modulator of each task. The resulting contrast images were taken up to the group-level, where we used correlations with the participant-specific percentage of low effort/value project chosen. In order to identify seed regions that are common to the truthtelling and control tasks we performed two conjunction analyses[5,6], one to identify an overlap between cost-related activations in the truthtelling and the effort task, and one to identify an overlap between cost-related activations in the truthtelling and the valuation task. The threshold was set to $p < 0.005$ for both contrasts in each of the conjunction analyses, thus including common voxels that showed significantly greater activation above this threshold.

Specificity PPI analysis. In order to investigate whether the connectivity pattern found for the truthtelling task (see main text) is specific for the truthtelling task and honesty-related values, we performed additional PPI analyses in order to compare the connectivity pattern of the truthtelling task with that of the effort task (the valuation task was not included due to lack of common voxels serving as seed; see Supplementary Results). The analyses were conducted similarly as described in the main text. Now, we used the voxels in the DLPFC and DMPFC that resulted from the conjunction analysis described above (and see Supplementary Results). Cost-related activity in these voxels correlated with the individual percentage of truthtelling as well as with the individual percentage of effort choice. Thus, the seed regions were based on activations common for both tasks. For each subject, the average time series was extracted from these overlapping DLPFC and DMPFC seed regions, which served as physiological regressors in two first-level general linear models. Moreover, the models included four psychological regressors (high cost and low cost for the truthtelling task, and high cost and low cost for the effort task), and four PPI regressors that were created by multiplying the time series with the

four psychological regressors, respectively. For each participant we computed the contrast between the PPI regressor of high vs. low cost in the truthtelling task and high vs. low cost in the effort task. The resulting contrast images were submitted to a second-level ANOVA (within-subject) that included honesty-related and effort-related (Supplementary Methods: Measurement of effort-related values) values as a covariate for the truthtelling and effort task, respectively. We searched for voxels in which honesty-related values more strongly correlated with functional connectivity in the truthtelling task than effort-related values in the effort task by contrasting the two task-specific covariates.

Supplementary Behavioral Results

Average behavior in control tasks and additional regression analyses. In the effort task participants chose the low effort option with similar frequencies to the truthtelling task (40.1% ± 5.0%, range 0 - 93.3%). We observed variation in their choices with respect to the economic cost of choosing the low effort option: 84.6% ± 4.8% in the zero cost condition, 52.5% ± 8.3% in the 1 CHF, 35.6% ± 7.7% in the 2 CHF, 17.3% ± 6.1% in the 3 CHF, and 10.6% ± 4.7% in the 4 CHF condition. In the valuation task participants chose the low value option less often than in the truthtelling task (18.8% ± 2.6%, range 0 - 60%). Note that the majority (72.2%) of the trials in which participants chose the low value option had zero cost (i.e., the low value option lead to the same payoff as the high value option): 67.9% ± 5.68% in the zero cost condition, 15.4% ± 5.8% in the 1 CHF, 8.3% ± 3.6% in the 2 CHF, 1.9% ± 0.8 in the 3 CHF, and 0.6% ± 0.4% in the 4 CHF condition. The mean switching point was 1.62 ± 0.25 CHF for the effort task and 0.58 ± 0.11 CHF for the valuation task.

We tested whether participants' choices differed as the economic costs of truthfulness changed by regressing the choices against the cost-level. This revealed significant effects of cost ($\beta = -0.95 \pm 0.13$ (mean ± SEM), $t = -7.29$, $p < 0.001$) on truthful decisions (fewer truthful decisions with increasing economic costs), suggesting that on average participants traded off the economic cost of telling the truth with its moral benefits. We observed similar effects for the two control tasks: the cost-level predicted whether or not participants chose the low effort/value option (effort task: $\beta = -1.02 \pm 0.15$, $t = -6.91$; both $p < 0.001$; valuation task: $\beta = -1.50 \pm 0.19$, $t = -8.06$).

Participants on average exhibited intermediate honesty-related moral values in their answers to the questionnaire (4.1 ± 0.2 (mean ± SEM), range 1.8 – 6.0). Without taking cost level into account, logistic regressions revealed no significant relations between moral values and choice behavior in any of the tasks (all $\beta < 0.25$, $t < 1.70$, $p > 0.09$).

Relation of honesty-related moral values to behavior in control tasks and to response times. In the truthtelling task, we observed a positive interaction between moral values and economic costs of truthfulness. We tested whether a similar interaction effect occurred for the two control tasks. In these tasks, too, the decisions the participants made as a CEO affected their compensation (see Supplementary Methods). Importantly, in all tasks the variable economic value difference between the two options was the same, but only the truthtelling task involved a trade-off with the motive of honesty. While there was no interaction effect at all for the effort task ($\beta = 0.12 \pm 0.13$, $t = 0.92$, $p = 0.36$; Supplementary Fig. S1A), in the valuation task participants with strong moral values chose the low value option less often in the zero cost condition ($\beta = 0.31 \pm 0.14$, $t = 2.16$, $p < 0.05$; Supplementary Fig. S1B). This interaction effect is quite different from the one in the truthtelling task (Fig. 1B), where participants with high moral values chose the truthful option more often in high cost conditions. Indeed, when excluding the zero cost condition (and thus including only trade-off conditions, i.e. situations in which there was an actual economic cost for telling the truth) the interaction effect for the valuation task disappeared completely ($\beta = 0.14 \pm 0.16$, $t = 0.88$, $p = 0.38$) but was still present, at least at trend level, for the truthtelling task ($\beta = 0.30 \pm 0.16$, $t = 1.91$, $p = 0.06$).

In further regression analyses we also tested whether response times in the truthtelling task were related to moral values and cost-levels. We observed neither a main effect of cost ($\beta = 7.49 \pm 5.07$, $t = 1.48$, $p = 0.15$) or moral values ($\beta = 13.56 \pm 15.18$, $t = 0.89$, $p = 0.38$) nor a cost x moral values interaction ($\beta = 1.73 \pm 5.79$, $t = 0.30$, $p = 0.77$). Moreover, we also found no effect of choice on response times in the truthtelling task ($\beta = -17.77 \pm 30.79$, $t = -0.58$, $p = 0.57$).

Control analyses with demographic variables and other individual-specific measures. We performed several additional analyses to test whether any of our demographic variables or individual-specific

measures is correlated with honesty-related values or change the cost-dependent relation of honesty-related value to behavior when added as control variables. All measures were collected together with our questionnaire for honesty-related values after the experiment in the scanner. The demographic variables were: Gender (0 = male; 1= female), age (completed years), employment (0 = No; 1 = Yes), monthly net income (amount in CHF), monthly net income of parents (amount in CHF). Additionally, we measured the following variables:

Impression management and self deception: We used the German version of the Deception Scales (PDS) of Paulhus[7,8] and measured individuals' tendencies to give socially desirable responses. The questionnaire contains two subscales; one measuring the tendency to deceive others (impression management) and one measuring the tendency to deceive oneself (self deception).

Altruistic concern: We asked participants the extent to which they believed that announcing 35 cents (dishonest option) had consequences for other stakeholders (-2 = hurting other stakeholders to +2 = not hurting other stakeholders). This variable was a relevant control for any altruistic preferences or fairness concerns of the participants (although in the context of the experiment there were no such consequences).

Valuation differences: Using the survey question of Miller and colleagues[9], we measured valuation differences, thus creating a proxy for marginal utility of money. The precise question is: *Please imagine that you find a CHF 50 bill on the street. It is impossible to identify the owner, and it is, therefore, completely acceptable and morally unobjectionable that you keep the CHF 50. Think about your average peer who earns about the same amount of money as you do, and is approximately equally wealthy. Would you say that, relative to this average peer, you benefit a lot more / more / equally / less / a lot less from this additional amount of money?* We assigned a value of 5 to ``a lot more" answers, and a value of 1 to ``a lot less" answers.

First, we tested whether any of the demographic or individual-specific variables correlate with our measure of honesty-related values. We neither found a correlation between honesty-related values and any of the demographic control variables (all $p > 0.12$), nor between honesty-related values and any of the other individual-specific measures (all $p > 0.22$).

Next, we tested whether any of these variables influence the cost-dependent relation of honesty-related values to behavior (see Results: Honesty-related values predict decisions in different cost situations) when added as control variables. We performed logistic regression analysis as described in the main text (Methods: Behavioral analysis), and added the demographic and other individual-specific measures as control variables. Even after controlling for these variables, we observe a positive interaction between honesty-related values and economic costs of truthfulness ($\beta = 0.31 \pm 0.14$, $t = 2.15$, $p < 0.05$). Thus, our measure of honesty-related values predicts cost-dependent choice over and above any of the demographic or other individual-specific variables.

Supplementary Neuroimaging Results

Choice-dependent neural activity. To identify neural activity that directly reflects honest behavior we compared trials in which participants actually decided to announce the true earnings to trials in which participants lied and engaged in earnings management. For this analysis we included twenty-one participants who showed both honest and dishonest behavior. The comparison of honest > dishonest decisions revealed increased activity in the temporal cortex (58, -40, -16; $t_{(20)} = 6.11$), parietal cortex (peak at 32, -68, 48; $t_{(20)} = 5.98$, and extending to TPJ at 46, -48, 32; $t_{(20)} = 5.32$), the DLPFC (40, 12, 44; $t_{(20)} = 5.62$), and the occipital lobe (-40; -84, -12; $t_{(20)} = 4.57$; all $p < 0.05$, whole-brain FWE cluster-level corrected; see Supplementary Figure S3 and Table S1). When lowering the threshold to p = 0.001 (uncorrected), we additionally found activation in the ACC and VLPFC. For the contrast of dishonest > honest decisions we found increased activation in the posterior cingulate cortex (-14, -40, 14; $t_{(20)} = 4.45$) and at a lower threshold (p = 0.001, uncorrected) in the anterior cingulate cortex (6, 34, -4; $t_{(20)} = 4.08$; see Supplementary Figure S3 and Table S1).

Control analyses: difficulty. We performed an additional analysis (Supplementary Methods: Measurement of effort-related values) to explore the possibility that the DLPFC and DMPFC activations from the seed identification analysis represent decision difficulty. Our aim was to investigate whether activity increase in the DLPFC and DMPFC with the percentage of truthtelling/low effort chosen is more strongly related to cost than to difficulty. To this end, we

performed for the truthtelling and effort task separately a second-level correlation analysis of the contrast PM cost vs. PM difficulty with the percentage of truthtelling and low effort chosen, respectively. Albeit at less stringent thresholds, we found in both tasks a stronger relation to cost than to difficulty in the DLPFC and DMPFC (truthtelling task: DLPFC: -28, 54, 28; $t_{(24)} = 4.90$; DMPFC: -8, 20, 44; $t_{(24)} = 4.88$; effort task: DLPFC: -30, 56, 12; $t_{(23)} = 4.28$; DMPFC: 6, 28, 32; $t_{(23)} = 3.60$, all $p < 0.001$, uncorrected), suggesting that the activity of these regions is explained by cost over and above choice difficulty.

Comparison between different types of honesty values. As described in the main text, our honesty values questionnaire assessed participants' reluctance to trade-off the moral value of honesty against economic incentives. Another way of assessing honesty-related moral values is examining subjective emotional reactions to violations of honesty[10–12]. Therefore, in addition to the questionnaire described earlier, we used a questionnaire that captured this emotional aspect of moral values with five items. For instance, we asked participants to indicate how blameworthy it is in their opinion when CEOs modify earnings reports. We wondered whether the modulation of DLPFC-IFG and DMPFC-IFG connectivity by honesty-related values is specific to our measurement of trade-off resistance. We therefore included the individual responses from emotional reactions questionnaire as a second covariate in the correlation analyses (note that in this study the emotional scale predicts resistance against economic costs weakly ($p = 0.12$)). We contrasted the trade-off reluctance with the emotional reaction scale and found that at less stringent thresholds both cost-dependent DLPFC-IFG and DMPFC-IFG couplings increased more strongly with trade-off resistance than with emotional reaction (DLPFC-IFG: -48, 26, 30; $t_{(29)} = 3.85$; DMPFC-IFG: -48,28,28; $t_{(29)} = 3.28$, both $p < 0.001$, uncorrected). These results suggest that in the domain of honesty, the prefrontal connectivity modulations are preferentially related to moral values that are expressed by trade-off reluctance rather than emotional reactions.

Control analyses: PPI with percentage of truthtelling and response times as covariates of no interest. We performed a control analysis to ensure that that the observed DLPFC-IFG and DMPFC-

IFG connectivity (see Results in the main text) is driven by honesty values rather than the percentage of truthtelling. Moreover, we also aimed to account for response times (remember that at the behavioral level there was neither a relation between cost-levels and response times nor between honesty value strength and response times; see above). We therefore asked whether the modulation of DLPFC-IFG and DMPFC-IFG connectivity by the strength of honesty-related values would still emerge when we also accounted for individual differences in the percentage of truthtelling and response times in the truthtelling task. We included percent truthtelling and mean response times as covariates of no interest in the correlation analyses and again found that both DLPFC-IFG and DMPFC-IFG coupling increased more in high than low cost conditions as honesty-related values increased (DLPFC-IFG: -46, 28, 30; $t_{(29)} = 4.83$; DMPFC-IFG: -44,28,28; $t_{(29)} = 5.51$, both $p < 0.05$, whole-brain FWE cluster-level corrected). Together, these results suggest that moral motives impact how core decision regions interact during decisions concerning honesty.

Specificity PPI analyses. As described in the main text, we found that in the truthtelling task both DLPFC and DMPFC showed stronger functional connectivity with the IFG in high cost conditions compared to low cost conditions as honesty-related values increased. One may ask whether the modulation of the DLPFC-IFG and DMPFC-IFG connectivity by honesty-related values is specific for the truthtelling task. To address this question, we performed additional PPI analyses (Supplementary Methods: Specificity PPI analysis) in which we compared the connectivity pattern of the truthtelling task with that of the control tasks. The analysis was based on the fact that in all tasks participants were confronted with the same economic cost-levels when choosing between the honest or low effort/value option on the one hand and the dishonest or high effort option on the other hand. Thus, the only additional component in the truthtelling task was the moral nature of the decision made. To do so, we first aimed to identify seed regions that are common for the truthtelling task and the effort or the valuation task. We therefore performed conjunction analyses by searching for brain regions where cost-dependent activation changed as a function of truthtelling and low effort choice on the one hand and truthtelling and low value choice on the other hand. For the conjunction between the truthtelling and effort task we found that with increasing cost of choosing the honest/low effort option,

participants show stronger activation in the DLPFC and DMPFC the more often they actually choose the honest/low effort option (DLPFC: -40, 56, 10; $t_{(60)} = 3.23$; DMPFC: 6, 26, 36; $t_{(60)} = 3.93$; both $p < 0.001$, uncorrected). Clusters in these two regions were used as seed regions for the specificity PPI analyses. We did not find any common activation between the truthtelling and the valuation task. We therefore did not perform any further analyses with the valuation task.

In order to identify voxels in which honesty-related values correlated with functional connectivity more strongly in the truthtelling task than effort-related values (Supplementary Methods: Measurement of effort-related values) in the effort task, we entered the contrast images resulting from the PPI analyses (truthelling high vs. low cost and effort high vs. low cost) into a second-level ANOVA and correlated them with honesty-related and effort-related values, respectively. For both seed regions this comparison revealed no significant effects, even when reducing the voxel-wise threshold to $p < 0.01$, uncorrected, suggesting little evidence for specificity of prefrontal coupling during honesty-related decisions as compared to effort-related decisions.

To assess whether monetary valuation would explain our data, we included marginal utility as a second covariate in our main analysis of cost-dependent relations between functional connectivity and honesty-related values. This revealed similar results for the correlation with honesty related values: DLPFC: -46, 28, 30; $t_{(30)} = 4.57$; DMPFC: -44, 28, 28; $t_{(30)} = 5.02$; $p < 0.05$, both whole-brain FWE cluster-level corrected. Moreover, a direct comparison between honesty related values and marginal utility identified similar regions, although at a slightly weaker threshold: DLPFC: -46, 28, 320; $t_{(30)} = 3.85$; DMPFC: -46, 26, 28; $t_{(30)} = 3.79$; both $p = 0.001$ uncorrected. These findings suggest that variation in monetary valuation does not explain our brain data. Moreover, they point to relative specificity with individual differences in honesty-related values as compared to individual differences in the value of money.

Supplementary Discussion

DLPFC-IFG and DMPFC-IFG connections are not specific to honesty-related decisions

Our results re-visit the question of whether moral decisions are represented by domain-specific or domain-general neural mechanisms[1,4]. Our findings suggest that individual differences in honesty-

related values predict the strength of DLPFC-IFG and DMPFC-IFG connectivity in the honesty task, but that these interactions are not specific for decisions concerning honesty. Thus, our data are in-line with domain-generality[2]. However, one possibility for proponents of domain-specificity to object might be that in the effort task participants could have perceived the decision to work more or less as a moral one because choosing the less effortful option could be considered as lazy. This would suggest that our findings are not specific to honesty-related moral decisions but that they rather reflect more general characteristics of moral decision making while maintaining domain-specificity at a higher level. Future studies should address this issue by investigating different types of moral decisions.

References

1. Greene, J. D. & Haidt, J. How (and where) does moral judgment work? *Trends Cogn. Sci.* **6,** 517–523 (2002).
2. Shenhav, A. & Greene, J. D. Moral judgments recruit domain-general valuation mechanisms to integrate representations of probability and magnitude. *Neuron* **67,** 667–677 (2010).
3. Tobler, P. N., Kalis, A. & Kalenscher, T. The role of moral utility in decision making: An interdisciplinary framework. *Cogn Affect Behav Neurosci* **8,** 390–401 (2008).
4. Mikhail, J. Universal moral grammar: Theory, evidence and the future. *Trends Cogn. Sci.* **11,** 143–152 (2007).
5. Friston, K. J., Penny, W. D. & Glaser, D. E. Conjunction revisited. *Neuroimage* **25,** 661–667 (2005).
6. Nichols, T., Brett, M., Andersson, J., Wager, T. & Poline, J.-B. Valid conjunction inference with the minimum statistic. *Neuroimage* **25,** 653–660 (2005).

7. Paulhus, D. L. Two-component models of socially desirable responding. *Journal of Personality and Social Psychology* **46,** 598–609 (1984).

8. Musch, J., Brockhaus, R. & Bröder, A. Ein Inventar zur Erfassung von zwei Faktoren sozialer Erwünschtheit. *Diagnostica* **48,** 121–129 (2002).

9. Miller, N., Wagner, A. F. & Zeckhauser, R. J. Solomonic separation: Risk decisions as productivity indicators. *J Risk Uncertain* **46,** 265–297 (2013).

10. Gibson, R., Tanner, C. & Wagner, A. Preferences for truthfulness: Heterogeneity among and within individuals. *Am. Econ. Rev.* **103,** 532–548 (2013).

11. Hanselmann, M. & Tanner, C. Taboos and conflicts in decision making: Sacred values, decision difficulty and emotions. *Judgm Decis Mak* **3,** 51–63 (2008).

12. Tetlock, P. E., Kristel, O. V., Beth, S., Green, M. C. & Lerner, J. S. The psychology of the unthinkable: Taboo trade-offs, forbidden base rates, and heretical counterfactuals. *Journal of Personality and Social Psychology* **78,** 853–870 (2000).

Supplementary Table S1. Brain regions exhibiting choice-related activation.

Brain region	*x*	*y*	*z*	*t*
**Honest vs. Dishonest**				
Middle temporal gyrus	58	-40	-16	6.11*
	-52	-36	-10	3.44
Superior parietal lobule	32	-68	48	5.98*
Inferior parietal lobule	-48	-48	50	4.88
Middle frontal gyrus / DLPFC	40	12	44	5.62*
	-42	32	36	3.95
Inferior occipital gyrus	-40	-84	-12	4.57*
Cuneus	-10	-96	-2	4.02
Superior frontal gyrus / DMPFC	6	30	50	4.28
Middle frontal gyrus /VLPFC	-32	52	-8	4.03
**Dishonest vs. Honest**				
Posterior cingulate cortex	-14	-50	14	4.45*
	14	-54	18	3.95
Anterior cingulate cortex	6	34	-4	4.08
	-6	32	-4	3.38

*Regions that survive whole-brain FWE correction at the cluster level ($p < 0.05$). Remaining activations have a p-value of $p < 0.001$, uncorrected. Coordinates are denoted by x, y, z (in mm; MNI space).

Supplementary Figure Legends

Figure S1. Trial structure of the control tasks. **(A)** In each trial of each task, participants first viewed a fixation cross for a variable ITI of 2 – 6 s followed by the presentation of a cue (1 s) that indicated which kind of task participants had to perform. The first, variable option was then shown for 3 s. In the effort task (top), the option for solving one problem was presented, and in the valuation task (bottom), the option for the low value project (project ZEM) was shown. Below the option the CEO compensation was indicated together with the corresponding participant payoff in parentheses. The payoff of the first options varied between 1 and 5 CHF. After an interstimulus interval (2 - 6 s) the second, constant option (5 CHF) was presented together with the first option. The second option consisted of performing five calculations in the effort task and the high value project in the valuation task (project XIR). Upon presentation of the second option, participants had 2 s to indicate their choice by performing a button press. When subjects pressed a button, the color of the written text on the screen changed from white to yellow to indicate that a response had been recorded.

Figure S2. Behavioral results for the effort and the valuation task. **(A)** In the effort task, participants with strong honesty-related values tended to choose the low value option more often than participants with weak honesty-related values ($\beta = 0.25$, $t = 1.70$, $p = 0.09$). Importantly, in contrast to the truthtelling task (Fig. 1B), in the effort task there was no interaction between costs and honesty-related values ($\beta = 0.12 \pm 0.13$, $t = 0.92$, $p = 0.36$). **(B)** In the valuation task, participants with strong moral values chose the low value option less often in the zero cost condition ($\beta = 0.31 \pm 0.14$, $t = 2.16$, $p < 0.05$). Note that this interaction effect is quite different from the one in the truthtelling task (Fig. 1B), where participants with high moral values chose the truthful option more often in high cost conditions.

Figure S3. Choice-dependent neural activity. Regions exhibiting increased activity for the contrast of honest vs. dishonest (A) and dishonest vs. honest (B).

Figure S4. (related to Fig. 3). Differential coupling between prefrontal regions in individuals with strong and weak honesty values. (A) DLPFC-IFG. (B) DMPFC-IFG. In both cases, connectivity was increased when honesty carried high rather than low costs in individuals with strong honesty values. The inverse pattern arose in individuals with weak honesty values, which explains why there are negative values in Fig. 3 (y-axis in Fig. 3: difference between high and low costs). When plotting connectivity separately for the two cost conditions and groups (this figure), coupling was positive. The group of participants was split into individuals above and below the median honesty value.

Figure S5. Example of an implausible connection model. The illustration shows an example of a model we excluded from the DCM analysis. In this model the driving input enters only the DLPFC and the modulation of connectivity is from the IFG to the DLPFC and the DMPFC but not from the DLPFC to the IFG. However, the modulation from the DLPFC to the IFG should be present if the information entering only the DLPFC is to exert any effect.

Figure S6. Bayesian model selection over all subjects, irrespective of their honesty-related values. The most likely model family (family 8 in Fig. 4A; exceedance probability = 0.43) was the one with a unidirectional connection from the IFG to the DLPFC and a reciprocal connection between the DMPFC and IFG (modulated by high cost). Thus, the winning model family when considering all participants is the same as when including only subjects with strong honesty-related values.

Fig. S1

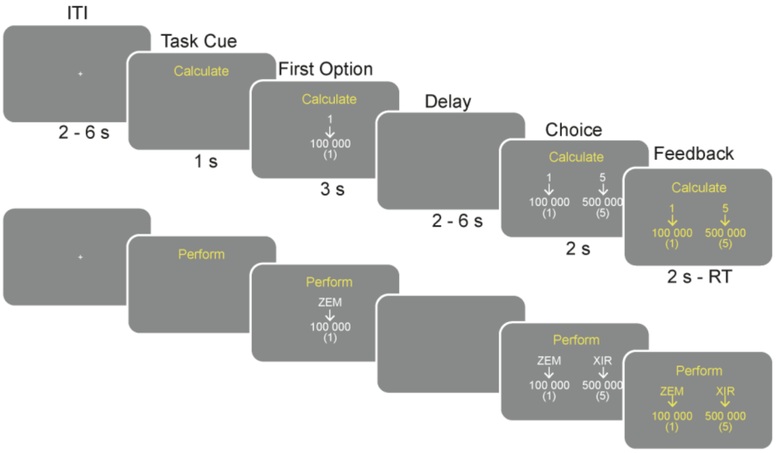

ITI
2 - 6 s
Task Cue
Calculate
1 s
First Option
Calculate
1
100 000
(1)
3 s
Delay
2 - 6 s
Choice
Calculate
1 5
100 000 500 000
(1) (5)
2 s
Feedback
Calculate
1 5
100 000 500 000
(1) (5)
2 s - RT
Perform
Perform
ZEM
100 000
(1)
Perform
ZEM XIR
100 000 500 000
(1) (5)
Perform
ZEM XIR
100 000 500 000
(1) (5)

Fig. S2

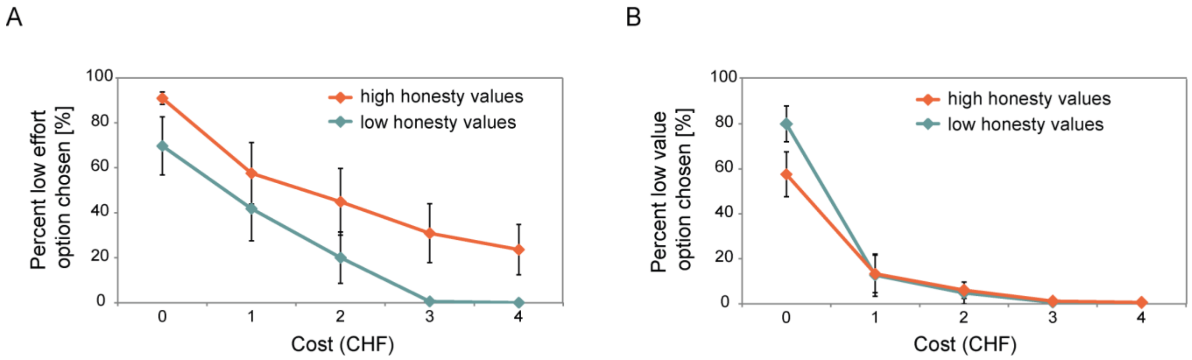

A
Percent low effort option chosen [%]
100
80
60
40
20
0
0
1
2
3
4
Cost (CHF)
high honesty values
low honesty values
B
Percent low value option chosen [%]
100
80
60
40
20
0
0
1
2
3
4
Cost (CHF)
high honesty values
low honesty values

Fig. S3

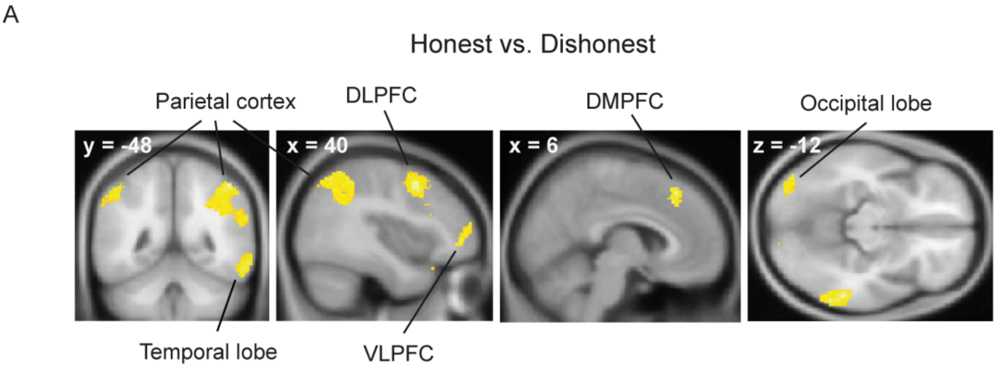
A
Honest vs. Dishonest
Parietal cortex
DLPFC
DMPFC
Occipital lobe
y = -48
x = 40
x = 6
z = -12
Temporal lobe
VLPFC
B
Dishonest vs. Honest
x = -10
x = 4
PCC
ACC

Fig. S4

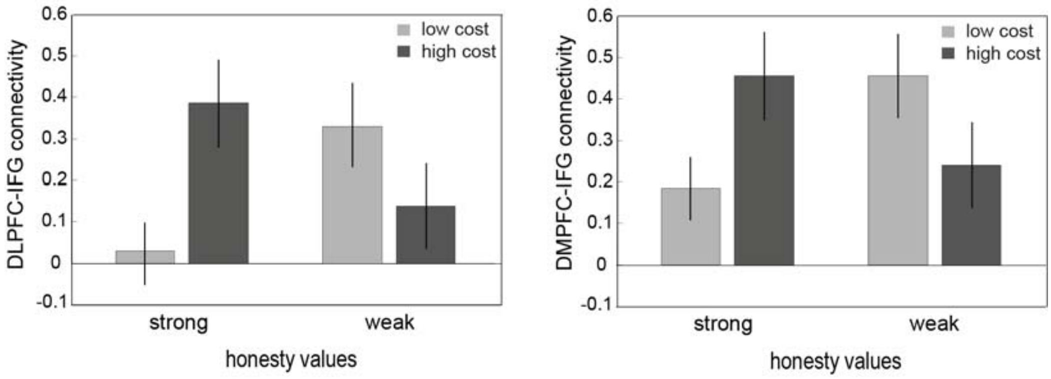
DLPFC-IFG connectivity
0.6
0.5
0.4
0.3
0.2
0.1
0
-0.1
low cost
high cost
strong
weak
honesty values
DMPFC-IFG connectivity
0.6
0.5
0.4
0.3
0.2
0.1
0
-0.1
low cost
high cost
strong
weak
honesty values

Fig. S5

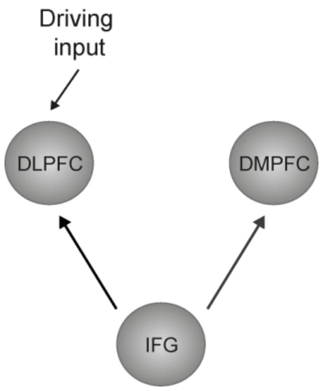
Driving input
DLPFC
DMPFC
IFG

Fig. S6

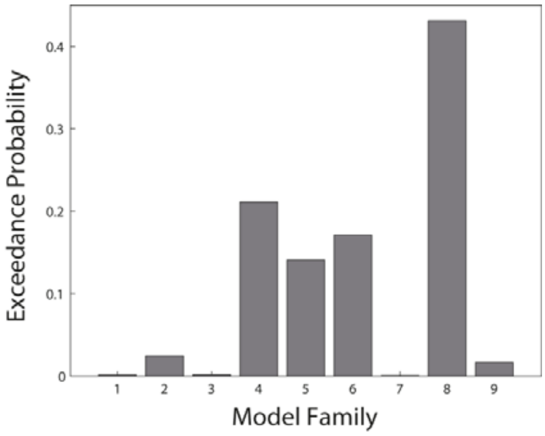
Exceedance Probability
Model Family
0
0.1
0.2
0.3
0.4
1
2
3
4
5
6
7
8
9
